# Non-native Pathway Engineering with CRISPRi for Carbon Dioxide Assimilation and Valued 5-Aminolevulinic Acid Synthesis in *Escherichia coli* Nissle

**DOI:** 10.1021/acssynbio.4c00318

**Published:** 2024-07-02

**Authors:** Sefli Sri Wahyu Effendi, I-Son Ng

**Affiliations:** Department of Chemical Engineering, National Cheng Kung University, Tainan 701, Taiwan

**Keywords:** *Escherichia coli* Nissle, CO_2_ fixation, Ribose 1,5-bisphosphate isomerase, RuBisCO, 5-ALA

## Abstract

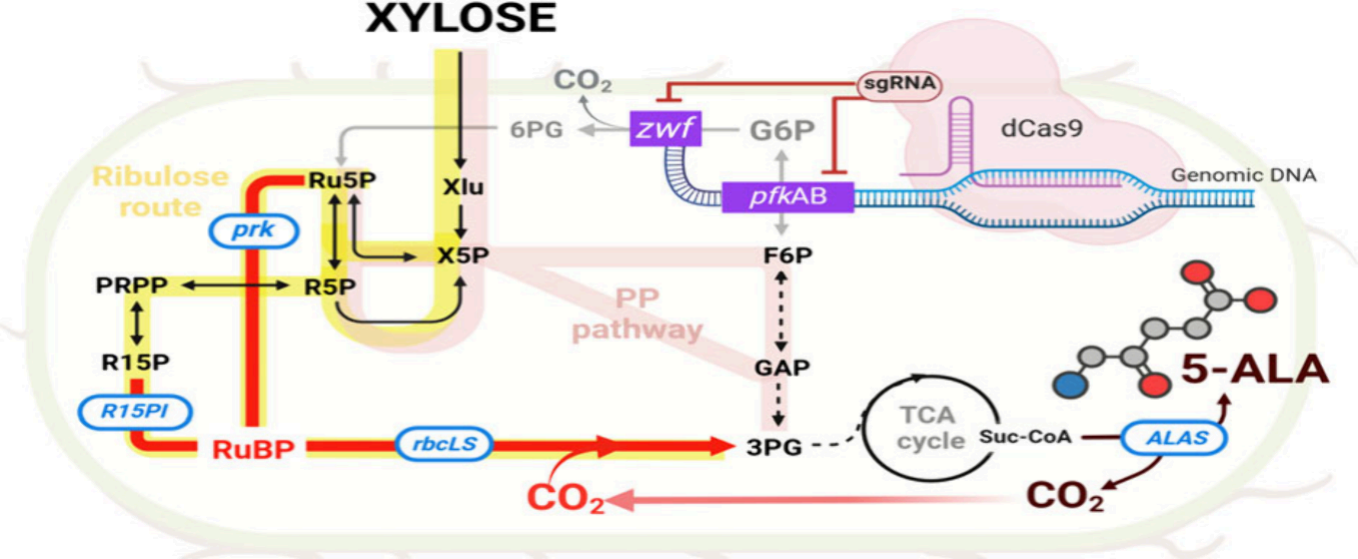

Carbon dioxide emission and acidification during chemical biosynthesis are critical challenges toward microbial cell factories’ sustainability and efficiency. Due to its acidophilic traits among workhorse lineages, the probiotic *Escherichia coli* Nissle (EcN) has emerged as a promising chemical bioproducer. However, EcN lacks a CO_2_-fixing system. Herein, EcN was equipped with a simultaneous CO_2_ fixation system and subsequently utilized to produce low-emission 5-aminolevulinic acid (5-ALA). Two different artificial CO_2_-assimilating pathways were reconstructed: the novel ribose-1,5-bisphosphate (R15P) route and the conventional ribulose-5-phosphate (Ru5P) route. CRISPRi was employed to target the *pfk*AB and *zwf* genes in order to redirect the carbon flux. As expected, the CRISPRi design successfully strengthened the CO_2_ fixation. The CO_2_-fixing route via R15P resulted in high biomass, while the engineered Ru5P route acquired the highest 5-ALA and suppressed the CO_2_ release by 77%. CO_2_ fixation during 5-ALA production in EcN was successfully synchronized through fine-tuning the non-native pathways with CRISPRi.

## Introduction

Growing concerns over environmental problems of traditional chemical production motivate the utilization of sustainable microbial cell factories (MCFs). However, the biological process of some chemicals involves a decarboxylation reaction and generates a CO_2_ byproduct.^1^ Releasing CO_2_ also would decrease extracellular pH due to the dissociation of carbonic acid.^2,3^ The alteration of acidic pH from pH 8.3 to pH 4.5 even inhibited bacterial growth up to 5-fold.^4,5^ For nonacidophilic strains, microorganisms require extra energy to maintain pH homeostasis by proton-translocating ATPase, thus reducing the MCF efficiency.^2,3^ Maintaining pH control during fermentation can mitigate acidification effects; however, dead zones in large-scale fermenters may hinder precise pH adjustment, thus posing challenges to accuracy.^6^ Therefore, incorporating an acidophilic strain with simultaneous CO_2_ fixation is a fantastic development to prevent and reduce CO_2_ emissions during chemical biosynthesis.^7^

Photosynthetic organisms are known for naturally accommodating CO_2_ assimilation. The Calvin–Benson–Bassham (CBB) cycle is an ancient CO_2_-fixing route that is highly regulated with ribulose-1,5-bisphosphate carboxylase-oxygenase (RuBisCO) and phosphoribulokinase (PRK).^8,9^ Despite this, the workhorse *Escherichia coli* has shown distinct traits over photoautotrophs such as five times faster growth, light independence, comprehensive genome databases, and sophisticated synthetic biology toolboxes for engineering metabolic pathways.^10,11^ Former works of engineered *E. coli* have successfully assimilated 35% CO_2_ into biomass via an artificial CBB cycle. The attempts were conducted by connecting the route from pentose phosphate (PP) into glycolysis along with utilizing formate as the electron source.^12^ Through adaptive evolution over 350 days, engineered *E. coli* utilized CO_2_ as the sole carbon source.^13^

Among *E. coli* lineages, Nissle 1917 (EcN) is the only probiotic *E. coli* that possesses acidophilic nature. The majority of EcN applications are related to living bacterial therapeutics and bowel resistance.^14^ Recently, EcN studies have been broadened as a safe chemical producer, thus leveraging its exploration for MCF. For instance, EcN has effectively produced a high titer bulk chemical of itaconic acid and high-value chemicals of *p*-coumaric acid, heparosan, aminobutyric acid, and 5-aminolevulinic acid (5-ALA).^15−19^ Of valuable chemicals and drugs, 5-ALA is a metabolic hub crucial for heme precursor and also has received approval from the FDA as a second-generation photosensitizer and photodynamic drug for the treatment of glioma.^20−22^ Biosynthesis of 5-ALA is majorly accomplished via the C4 pathway by overexpressing heterologous ALA synthase (ALAS), and CO_2_ is a definitive side product.^20,21^ Previous works of 5-ALA production from glucose have been integrated with the CO_2_ biomitigation system by co-overexpressing RuBisCO and PRK.^23,24^ However, rerouting flux from glucose into Ru5P would release additional CO_2_, indicating that CO_2_ assimilation from chemical biosynthesis has not been effectively executed.^12,25^ Moreover, as the strain used was *E. coli* BL21 or K-12, manipulating the acetate pathway was necessary to hinder a high acetate accumulation and maintain strain durability.^23,24^ Hence, harnessing the acidophilic nature of EcN to express the CO_2_-fixing system and fine-tuning its artificial pathway are outright solutions for assimilating CO_2_ emission during 5-ALA production.

Taken together, this study exploited two different CO_2_ assimilation pathways within the ribulose route via R15P and Ru5P and then connected them with a RuBisCO shunt. The common CO_2_ fixation route from Ru5P into 3PG was directed using PRK and RuBisCO from *Synechococcus elongatus* PCC6301 (denoted as PR).^26^ Another novel route between R15P and 3PG was bridged by synergizing R15P isomerase (R15PI) from *Thermococcus kodakarensis*(27) and RuBisCO from *S. elongatus* PCC6301 (denoted as RR). Subsequently, the function and efficiency of both CO_2_-fixing systems were evaluated for assimilating the release of CO_2_ during 5-ALA production. To strengthen the CO_2_-fixing pathway, a carbon flux was derived from xylose instead of glucose and further manipulated by using CRISPRi. Different levels of CO_2_ were also supplemented into the system. Finally, the efficiency of both CO_2_-fixing routes was examined in terms of biomass, 5-ALA production, and the CO_2_ assimilation capabilities.

## Results and Discussion

### Genetic Design of Artificial CO_2_-Assimilating Pathways via Ribulose Route

**Figure 1:**
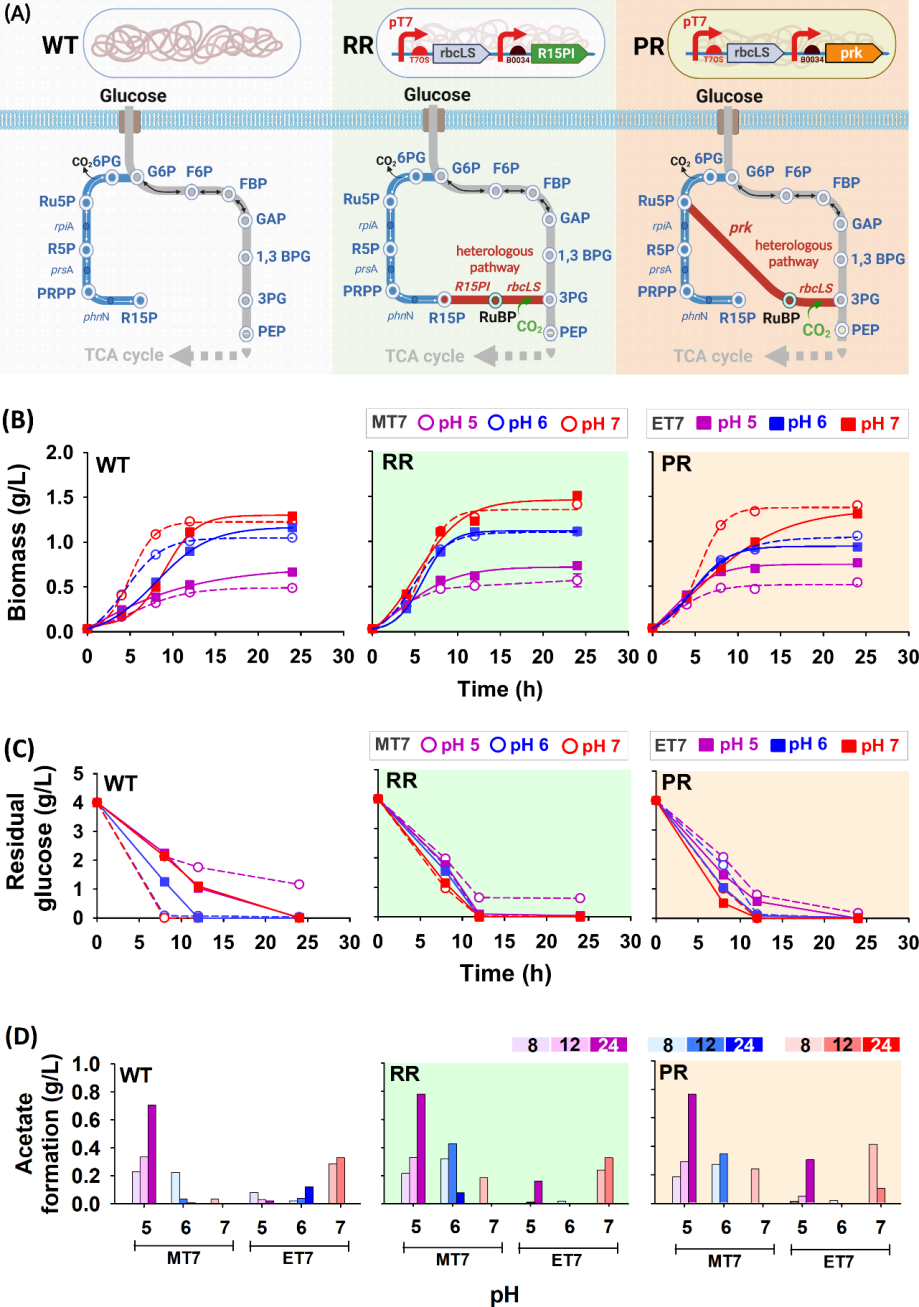
Reconstitution of two different non-native CO_2_ assimilation pathways in *Escherichia coli*. (A) Native pathway from glycolysis to the TCA cycle (left); artificial CO_2_-assimilating pathway in *E. coli* by harboring R15P isomerase (R15PI) from *Thermococcus kodakarensis* and RuBisCO (*rbcLS* gene) from *Synechococcus elongatus* PCC7942 (i.e., RR plasmid, middle); phosphoribokinase (*prk* gene) and RuBisCO (*rbcLS* gene) from *S. elongatus* PCC7942 under T7 regulations (i.e., PR plasmid, right). Time course of (B) biomass, (C) residual glucose consumption, and (D) acetate formation during culture of recombinant *E. coli* MT7 and ET7 at 8, 12, and 24 h. The strains were cultured in the glucose-based minimal medium (4 g/L) using a baffled flask in different pH cultures (pH 5, 6, 7) and 37 °C. Wild-type (WT) of T7RNAP-equipped MG1655 and EcN strains, denoted as MT7 and ET7. White, green, and orange backgrounds indicate MT7 or ET7 expressing empty plasmid, RR, and PR. (Metabolite abbreviations: G6P, glucose-6-phosphate; F6P, fructose-6-phosphate; FBP, fructose-1,6-biphosphate; GAP, glyceraldehyde 3-phosphate; 1,3 BPG, 1,3-bisphosphoglycerate; 3PG, 3-phosphoglycerate; PEP, phosphoenolpyruvate; 6PG, 6-phosphogluconate; Ru5P, ribulose-5-phosphate; R5P, ribose-5-phosphate; PRPP, 5-phospho-d-ribosyl-α-1-pyrophosphate; R15P, ribose 1,5-bisphosphate, RuBP: ribulose 1,5-bisphosphate, CO_2_: carbon dioxide).

An artificial CO_2_-fixing pathway in EcN was reconstructed at the downstream of the ribulose route to provide an additional carboxylation step, as in former works.^23,24^ Two different routes, R15P and Ru5P, were added to connect the pentose phosphate (PP) pathway to 3-phosphoglycerate (3PG), which is an intermediate in glycolysis. The first and second schemes were designated as RR and PR, respectively (Figure 1A). To reveal whether EcN has a distinguished profile toward the non-native CBB pathways, *E. coli* MG1655 was selected as a comparative host. EcN and MG1655 were equipped with T7 RNA polymerase (T7RNAP), yielding MT7 and ET7, for controlling CO_2_-fixing genes under the T7 promoter. Six recombinant strains were cultured in the glucose-based medium at pH 5, 6, and 7. At pH 5, all recombinant ET7 grew faster than MT7 strains even though the pH culture of ET7 was slightly lower than MT7 strains, confirming the acid tolerance of EcN (Figure S1). At pH 6 and 7, recombinant MT7 and ET7 showed a contrast profile. ET7 would have rapid growth by expressing the RR plasmid, while MT7 showed a similar finding by harboring PR genes (Figure 1B). The natural mutations of the R15P-producing gene (*phn*N) in EcN led to ET7 having better implementation toward the RR route than the MT7 strain (Figure S2).

In terms of glucose and acetate profiles, MT7 has a slow glucose consumption rate, which could be reasoned for its decelerated growth. Moreover, MT7 would accumulate a higher amount of acetate than ET7, except at pH 7. Such findings emphasized that this environment is unfavorable for EcN and limits the acetate-utilizing capability. Despite this, the acetate accumulation would be utilized as a secondary carbon source after depleting glucose (Figure 1C,D).^28,29^ Overall, all strains acquired the highest biomass at pH 7; yet, the effect of harboring CO_2_-fixing strains was intangibly observed from the growth profile. Hence, further evaluation was directly conducted for assimilating the release of CO_2_ from 5-ALA production.

### Comparative Efficiency of CO_2_-Fixing Strains for 5-ALA Production

**Figure 2:**
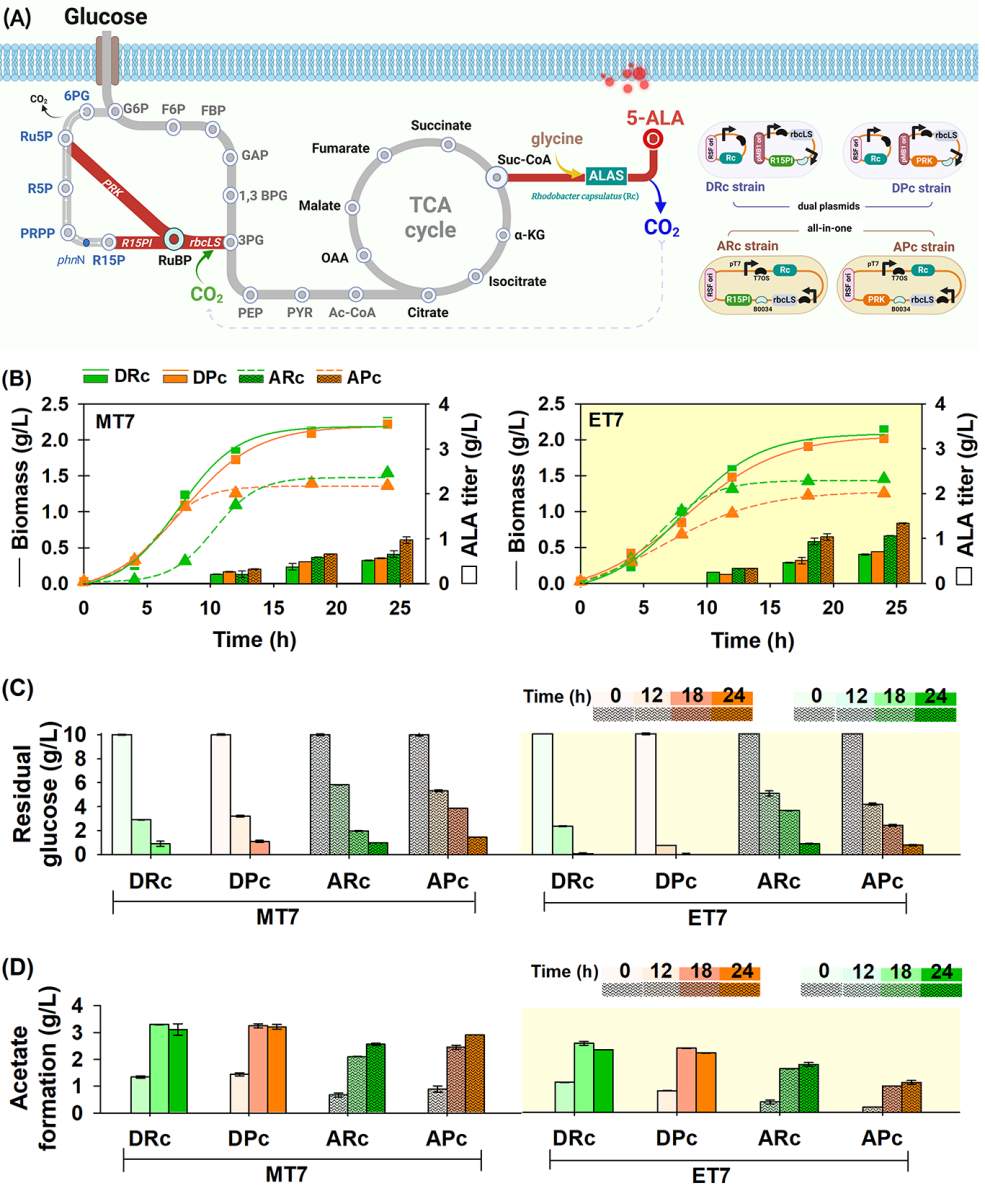
Functional comparison of overexpressing RR and PR in MT7 and ET7 strains for low-carbon-footprint 5-ALA production. (A) Metabolic pathway of low-carbon-footprint 5-ALA production in *E. coli* along with the genetic design of coupling CO_2_-fixing gene and ALA synthase (ALAS) from *R. capsulatus*. The recombinant expression was regulated under the T7 promoter and designated in two different systems of dual plasmids (“D”) and all-in-one plasmid (“A”). DRc and ARc are dual plasmids and all-in-one designs of R15PI-RuBisCO-Rc genes, respectively, while DPc and APc are dual plasmids and all-in-one design of PRK-RuBisCO-Rc genes, respectively. (B) Time course of biomass and ALA accumulation, MT7 on left and ET7 on right. (C) Residual glucose consumption and (D) acetate accumulation of recombinant MT7 (left) and ET7 (right) strains. The cells were cultured in 10 g/L glucose-based minimal medium pH 7 using a baffled flask at 37 °C. Glycine as cosubstrate was added *in vitro*. (Metabolite abbreviations: PYR, pyruvate; Ac-CoA, acetyl-CoA; a-KG, alpha-ketoglutarate; Suc-CoA, succinyl-CoA; OAA, oxaloacetate; 5-ALA, 5-aminolevulinic acid; TCA cycle: the citric acid cycle)

The functional application of an engineered CO_2_-fixing strain was examined for recycling the CO_2_ emission from 5-ALA production. The CO_2_-fixing system in the upstream pathway was expected to take up CO_2_ release and then increase the carbon flux in the downstream pathway. Eventually, this scenario might impact in improving of either biomass or target product from the overexpressed design (5-ALA in this study). To produce 5-ALA via the C4 pathway, a highly active ALAS from heterologous *Rhodobacter capsulatus* (Rc) was involved.^24^ The coexpression of Rc and CO_2_-fixing genes was designated into two systems of dual plasmids (DRc and DPc) and an all-in-one plasmid (ARc and APc) (Figure 2A). The evaluation was carried out in terms of biomass, 5-ALA titer, and metabolite profile. After 24 h of culture, both *E. coli* strains harboring the all-in-one design (ARc and APc) exhibited lower biomass than that of the coupled dual-plasmid system (DRc and DPc). The highest biomass, reaching 2.23 and 2.14 g/L, was acquired using DRc in MT7 and ET7 strains, respectively. Aside from lower biomass, the all-in-one strains displayed a relatively higher 5-ALA titer than the dual plasmid, with a remarkable titer of 1.52 g/L achieved using APc in ET7 (Figure 2B). Such behavior reflected a trade-off between biomass and metabolites. Due to using two strong T7 promoters, expressing CO_2_-fixing genes on dual plasmids might hinder Rc capability, thereby limiting 5-ALA production. In contrast, the all-in-one design controlled the CO_2_-fixing genes under a single, later T7 promoter, ensuring sufficient orthogonality of the T7 RNA polymerase for Rc overexpression. However, compared to the use of a single Rc gene, coexpressing Rc with CO_2_-fixing genes acquired a lower 5-ALA titer than Rc alone (i.e., 1.64 g/L). A sole Rc expression also achieved higher biomass up to 2.8 g/L, surpassing strains harboring RR or PR with a maximum biomass of 2.3 g/L, as shown in Figure S3. From the metabolic analysis, the dual plasmids and all-in-one design of MT7 strains have comparable glucose utilization, while dual plasmids in ET7 completely consumed glucose within 18 h (Figure 2C). Acetate was also highly accumulated in the dual-plasmid system (i.e., DRc and DPc), especially in MT7, reflecting a reason for limited 5-ALA production (Figure 2D).

Contrary to previous studies, the low 5-ALA titer and biomass in the strain harboring CO_2_-fixing genes were somewhat unexpected. This finding might correspond to the decarboxylation from 6PG to Ru5P, thus causing insufficient carbon flux in the RuBisCO pathway. Former studies also have reported that carbon flux from glucose is primarily directed into the main branch of glycolysis under both aerobic and anaerobic conditions.^25,30,31^ Hence, the enrichment of carbon flux in the ribulose-related pathway is critical to reveal the significant function of the CO_2_-fixing genes.

### Fine-Tuning Carbon Flux Using CRISPRi

**Figure 3:**
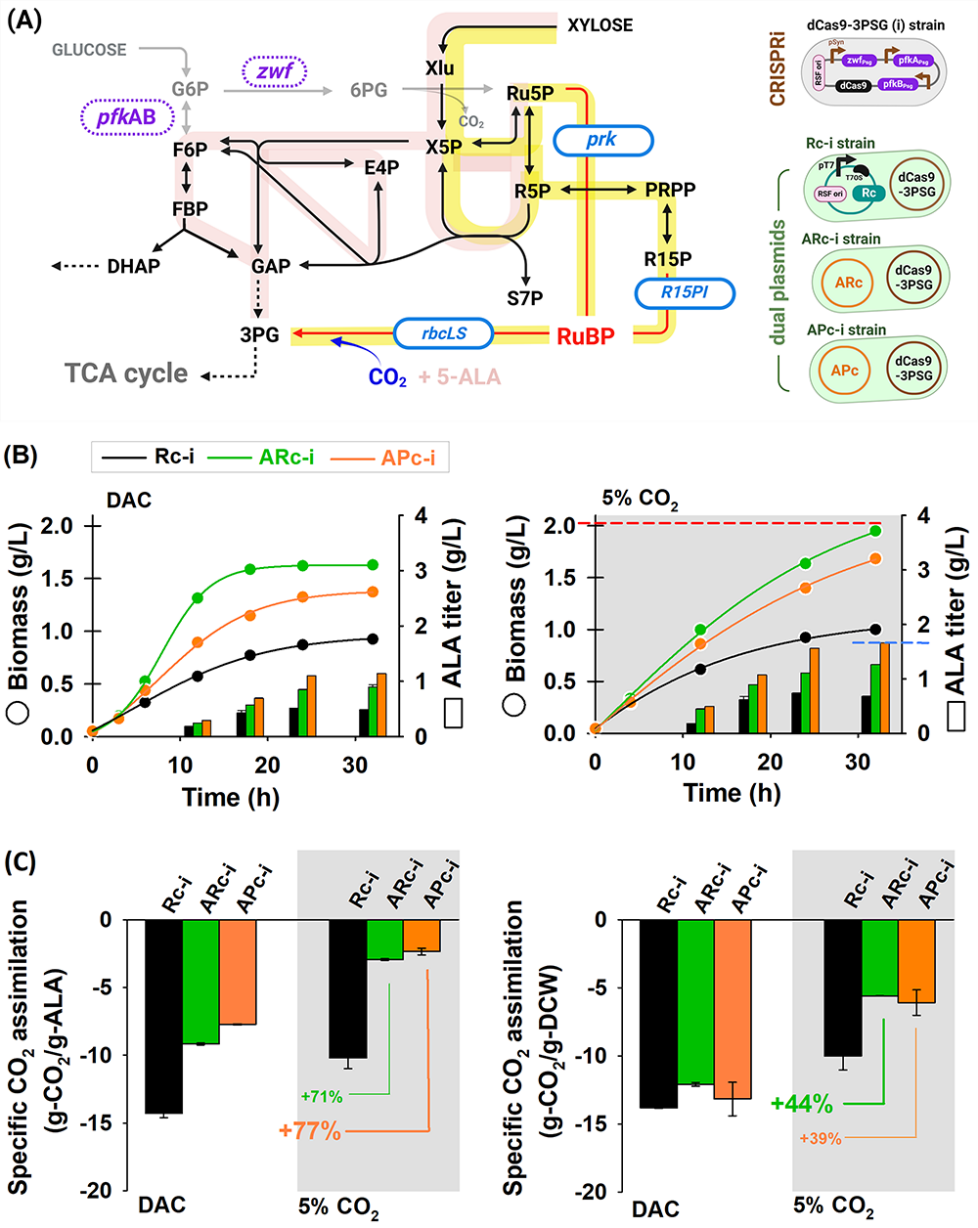
Design of CRISPRi for redirecting carbon flux from xylose to the CO_**2**_-fixing pathways for strengthening CO_2_ recycle and improving 5-ALA production. (A) Metabolic pathway of 5-ALA production using xylose and flux redirection through CRISPRi targeting *pfkAB* and *zwf* genes (i.e., dCas9–3PSG, denoted as “i”). Pink and yellow highlights indicate the native and the extra artificial route from CO_2_-fixing genes toward xylose utilization. All recombinant *E. coli* ET7 strains harbor dual plasmids: one is CRISPRi, and the other is plasmid Rc for Rc-i, plasmid RR for ARc-i, or plasmid PR for APc-i. The cells were cultured in 10 g/L xylose-based minimal medium pH 7 using a baffled flask as direct CO_2_ capture from air (i.e., DAC) and bioreactor with 5% CO_2_ supply at 37 °C, respectively. (B) Time course of biomass and 5-ALA during culture with DAC and 5% CO_2_ supply. Dashed red and blue lines indicate the highest biomass and 5-ALA acquired using ARc-i and APc-i, respectively. (C) CO_2_ assimilation capability in terms of g-CO_2_/g-5-ALA and g-CO_2_/g-DCW at 32 h. The percentage values represent remarkable CO_2_ suppression in each corresponding term. (Metabolite abbreviations: Xlu, xylulose; S7P, seudoheptulose-7-phosphate; E4P, erythrose-4-phosphate; DHAP, dihydroxyacetone phosphate. Purple for knocked-down and blue for overexpressed genes.)

Due to having an adjacent route with the ribulose-related pathway,^13,25,30,31^ xylose was chosen to disclose the distinguished nature of RR and PR. Moreover, to enhance flux toward the RuBisCO pathway, *zwf*, *pfk*A, and *pfk*B genes were knocked down, avoiding carbon loss (i.e., CO_2_ release) (Figure 3A).^25,29^ CRISPRi-based gene regulation was chosen due to its high efficiency and flexibility in multiple targeting specific loci in the genome. Additionally, CRISPRi requires only one protein, dCas9, to interfere with gene transcription by allosterically binding to the target gene.^32^ The effect of CRISPRi design was evaluated by coexpressing the sgRNA plasmid with the CO_2_-fixing genes in glucose- and xylose-based mediums. In the glucose medium, strains imposed on the CRISPRi system showed strong cell burden and limited growth. Conversely, such a design could stimulate the growth in the xylose medium, indicating that flux redirection was successfully executed (Figure S4). The effect of CRISPRi on the CO_2_-fixing strains was also evaluated in a xylose-based medium at 5% CO_2_ supply. The result showed a consistent behavior where coupling CRISPRi would have rapid growth (Figure S5).

Taken together, the Rc gene, the CO_2_-fixing genes, and the CRISPRi plasmid were incorporated, yielding Rc-i, ARc-i, and APc-i. Three recombinant ET7 strains were functionally evaluated for 5-ALA production from xylose with a direct air capture (DAC) of 420 ppm and 5% CO_2_ supply. As expected, the current design showed major impacts of RR or PR presence toward cell growth and 5-ALA. The fastest growth and highest biomass were performed by the ARc-i strain. Supplying 5% CO_2_ showed significant assistance that allowed the engineered CO_2_-fixing strains to continue growing (Figure 3B). In terms of 5-ALA production, by supplying 5% CO_2_, the APc-i strain achieved the highest 5-ALA of 1.66 g/L after 32 h. The findings presented trade-off phenomena in which obtaining a higher biomass would limit the strain in acquiring 5-ALA. At both CO_2_ levels, a single Rc yielded the lowest biomass and 5-ALA. It was caused by flux restriction from the CRISPRi design and the lack of a CO_2_-fixing pathway, which corresponded to a higher remnant xylose (Table S1).

Furthermore, the CO_2_ assimilation capability of recombinant strains under varied conditions was quantified according to the mass balance analysis. The detailed calculations are presented in Tables S1–S3. Coupling with CO_2_-fixing genes successfully suppressed CO_2_ emissions in terms of either 5-ALA titer or biomass when compared with a sole Rc. Corresponding to 5-ALA production, the APc-i strain performed a notable suppression of 77% by assimilating CO_2_ release from −10.57 g-CO_2_/g-5-ALA into −2.42 g-CO_2_/g-5-ALA). Interestingly, the CO_2_ assimilation under 5% CO_2_ inlet was detected to be higher than that in DAC. These results suggested that both strains could capture CO_2_ as a cosubstrate and correlated with higher remnant xylose (Table S1). According to the dry cell weight (DCW), a remarkable suppression of 44% was acquired using the ARc-i strain, which could assimilate CO_2_ from −10.0 g-CO_2_/g-DCW into −5.60 g-CO_2_/g-DCW. A high CO_2_ suppression toward 5-ALA using APc-i might be related to the short CO_2_-fixing shunt from Ru5P into 3PG. An implicit assumption is that carbon flux via the PR route could promptly coincide with overexpression of the Rc gene and the *in vitro* supply of glycine as a cosubstrate. Meanwhile, the RR design has a longer CO_2_-fixing shunt and interconnection with 3PG. Thus, it might retard the carbon flux, and thereby the directed flux into 5-ALA production did not equal the glycine supply.

To summarize, the reconstruction of two different CO_2_-fixing pathways has been successfully applied in EcN and *E. coli* MG1655 as a reference. Natural mutations in *phn*N gene allowed EcN to perform a better CO_2_ assimilation via the R15P route than MG1655. The functional efficiency of the CO_2_-fixing pathway via R15P resembled that of the conventional Ru5P route. Further optimizations focused on flux enhancement toward the CO_2_-fixing pathway. Adopting CRISPRi for flux redirection from xylose to the CO_2_ assimilation pathway was the critical key to efficiently utilizing CO_2_-fixing genes. Through this fine-tuned design, engineered EcN could achieve high biomass, 5-ALA, and CO_2_ assimilation capability. This study attempted the comparative analysis of two non-native CO_2_-fixing routes from the ribulose-related pathway and extended the promising potential of EcN as a low-carbon-featuring chemical producer.

## Materials and Methods

### Strains, Plasmids, Primers, and Media

All of the strains, plasmids, primers, and gene sequences are given in Table S4. *E. coli* DH5α was used for regular cloning, while T7RNAP-equipped MG1655 and EcN (MT7 and ET7) were used for functional tests. Luria–Bertani (LB) medium was used for preculture, while the MM9 medium (12.8 g/L Na_2_HPO_4_, 3 g/L KH_2_PO_4_, 0.5 g/L NaCl, 1 g/L NH_4_Cl, 0.24 g/L MgSO_4_, 0.011 g/L CaCl_2_) with 10 g/L glucose or xylose was used in the CO_2_ assimilation and 5-ALA production.^23^ The antibiotics at concentrations of 100 mg/L ampicillin, 50 mg/L kanamycin, and 25 mg/L chloramphenicol were added, while 0.1 mM IPTG was used for induction.

### Plasmid Construction

The natural R15PI gene (NCBI reference sequence ID: WP_011249140.1), PRK, and RuBisCO (*rbcLS*)^22^ were codon-optimized and synthesized from IDT (Table S4). The gene was amplified by PCR and cloned into the opted backbone. For the CRISPRi system, the design followed the previous study with medium copy number.^26^ After digestion by using the same restriction sites, the ligation process was carried out at 22 °C for 90 min, and the ligated plasmids were further transformed into *E. coli* DH5α using a heat shock method at 42 °C for 90 s with a recovery time for 1 h at 37 °C and 200 rpm. Recombinant colonies were grown on LB plates with appropriate antibiotics overnight and further confirmed by enzyme digestion. The positive plasmid was transformed into the expression strains using the heat shock method as aforementioned.

### Culture Conditions

The recombinant cells were cultured aerobically in a 250 mL baffled flask with 30 mL of glucose-based MM9 medium without CO_2_ supply.^23^ The whole culturing process was placed at 37 °C with shaking at 200 rpm. After optimizing the carbon flux using xylose-based MM9 medium and CRISPRi design, the cells were cultured in two different devices as (i) a 250 mL baffled flask with 30 mL medium and direct CO_2_ capture from the air (DAC) and (ii) a 100 mL Photon Systems Instruments (PSI) cultivator with 80 mL of medium and 5% CO_2_ supply (i.e., a flow rate of 40 mL/min).^26^ The co-precursors and cofactors of 5-ALA production such as 3 g/L glycine, 1 g/L succinate, 30 μM PLP, and 0.1 g/L ferric citrate were added to the culture when the cell density OD_600_ reached 0.6.

### Calculation of the CO_2_ Assimilation Capability by Element Analysis

The cells at 32 h were collected by centrifuging at 10000*g* for 10 min and washed with ddH_2_O thrice to define the relationship between DCW and the final point of OD_600_. The carbon content in DCW was analyzed using elemental analysis (UNICUBE, Germany) to obtain the amount of C_Biomass_. On the other hand, C_metabolites_ in and out represent the total carbon input and after culture, including remnant carbon and accumulated compounds. C_metabolites_ were determined by calculating the carbon content in 5-ALA and metabolites, which were measured using HPLC. The carbon coefficient from xylose is 0.40, while coefficients of lactate, succinate, acetate, 5-ALA, and CO_2_ are 0.40, 0.41, 0.4, 0.46, and 0.27, respectively. Afterward, specific CO_2_ assimilation capability was calculated using the below equation:^26^

Specific CO_2_ assimilation capability (g-CO_2_/g-5-ALA or g-CO_2_/g-DCW)
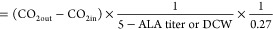

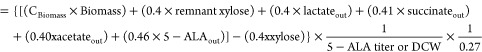


### Analysis of 5-ALA Production

The quantification of 5-ALA was performed using Ehrlich’s reagent, which consists of 0.1 g/mL *p*-dimethylamino benzaldehyde (DMAB) and 16% (v/v) hypochlorous acid in glacial acetic acid. Meanwhile, the sample reaction was prepared by mixing 0.5 mL of the sample or standard solution, 0.5 mL of 1 M acetate buffer (pH 4.6), and 0.1 mL of acetylacetone, followed by heating at 100 °C for 10 min. Afterward, 0.1 mL of the mixture and 0.1 mL of freshly prepared modified Ehrlich’s reagent were mixed in the 96-well plate and incubated in the dark for 10 min. Afterward, the samples were measured using a spectrophotometer (SpectraMax 340, Molecular, Devices, USA) with absorbance at 553 nm.^26^

### Metabolites Analysis Using HPLC

The supernatant was collected from the culture at the desired time and filtered with a 0.22 μM filter. Afterward, the sample was subjected to HPLC (Hitachi, Japan) analysis, which was equipped with ICSep COREGEL-87H3 (Transgenomic, USA) and a refractive index detector. The column temperature was set at 70 °C, and the mobile phase was 0.008 N H_2_SO_4_ with a flow rate of 0.4 mL/min.

## Supporting Information Available

Information on *phnN* amino acids sequence and its mRNA level; metabolite profile of recombinant *E. coli* MT7 and ET7; time course of growth and 5-ALA production using a sole Rc strain; growth profile of recombinant ET7 strains after knocking down *pfk*AB and *zwf* gene using CRISPRi in varied conditions; table listing the plasmids, primers, DNA sequence, and the quantification of CO_2_ assimilation (PDF)
