# Supplementary material for: Non-native Pathway Engineering with CRISPRi for Carbon Dioxide Assimilation and Valued 5-Aminolevulinic Acid Synthesis in Escherichia coli Nissle
Source: ACS Synth Biol. 2024 Jul 2;13(7):2038–44. doi: 10.1021/acssynbio.4c00318 (PMC11264323; doi:10.1021/acssynbio.4c00318)
Supplement: Supplementary file 1 — sb4c00318_si_001.pdf [file sb4c00318_si_001.pdf]

Letter to ***ACS Synthetic Biology***

Supporting Information

Non-native Pathway Engineering with CRISPRi for Carbon Dioxide Assimilation and Valued 5-Aminolevulinic Acid Synthesis in *Escherichia coli* Nissle

Sefli Sri Wahyu Effendi, I-Son Ng*

Department of Chemical Engineering, National Cheng Kung University, Tainan 701, Taiwan

*Corresponding author: Prof. I-Son Ng

Tel: +886-62757575-62648; Fax: +886-62344496

E-mail: yswu@mail.ncku.edu.tw

Dr. I-Son Ng ORCID: 0000-0003-1659-5814

Dr. Sefli Sri Wahyu Effendi ORCID: 0000-0003-1766-544X

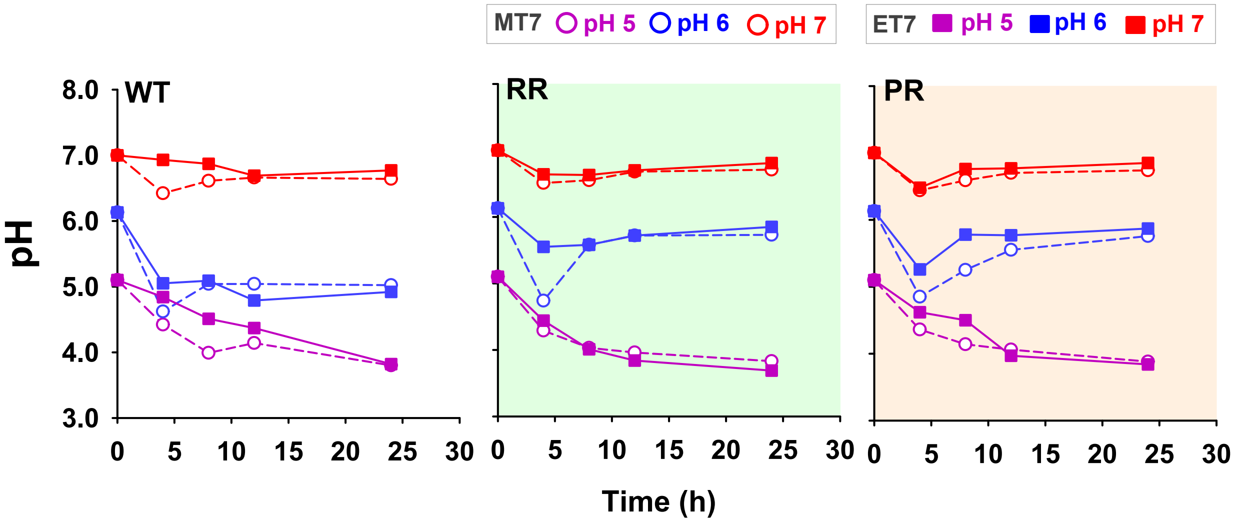

Figure S1 pH profiles of recombinant *E. coli* strains including wildtype (WT) of T7RNAP-equipped MG1655 and EcN strains, denoted as MT7 and ET7, RR and PR during the culture in the glucose-based minimal medium with initial pH 5, 6, and 7 at 37°C.

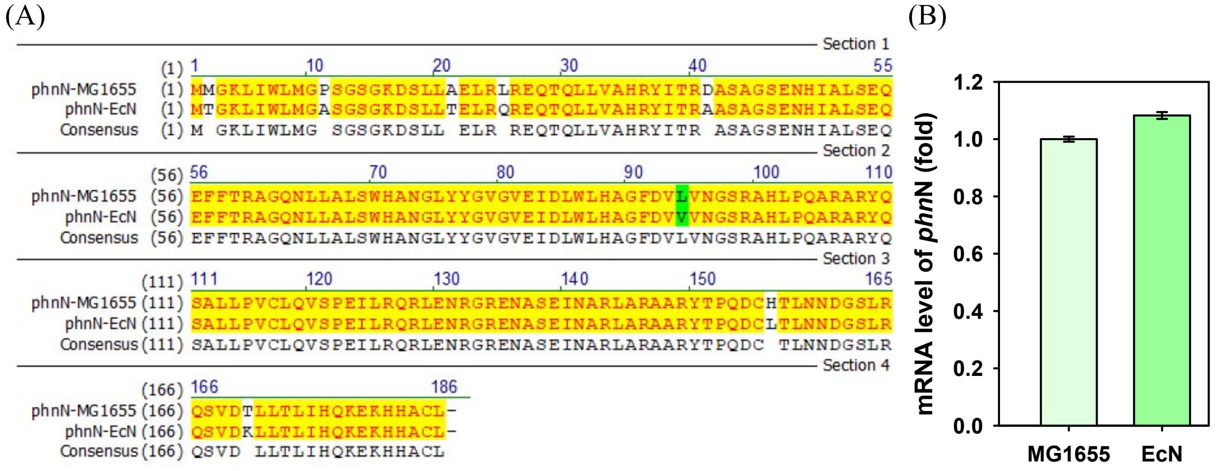

Figure S2 (A) Comparison of the amino acid sequence of *phn*N gene between MG1655 and EcN. Red lines indicate the conserved active sites of *phn*N gene. The green highlight and white colors represent the consensus and distinguished mutation of amino acids. **(B)** mRNA level of phnN gene in wildtype MG1655 and EcN. The transcription analysis was conducted by using samples after 12 h culture in the glucose-based medium.

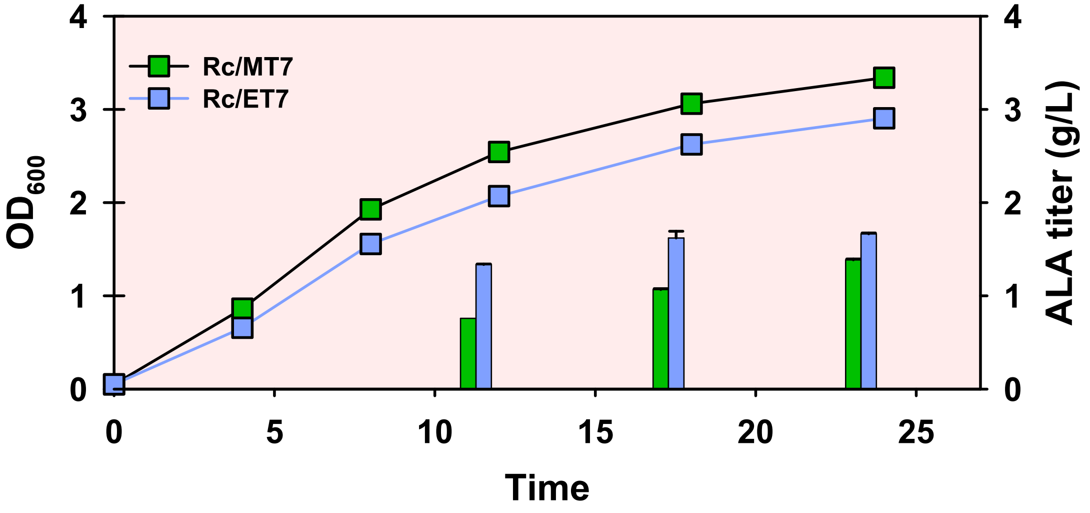

Figure S3 Time course of cell growth and 5-ALA production in a sole Rc-expressed strain. Strains were cultured using baffled flasks at 37°C**.**

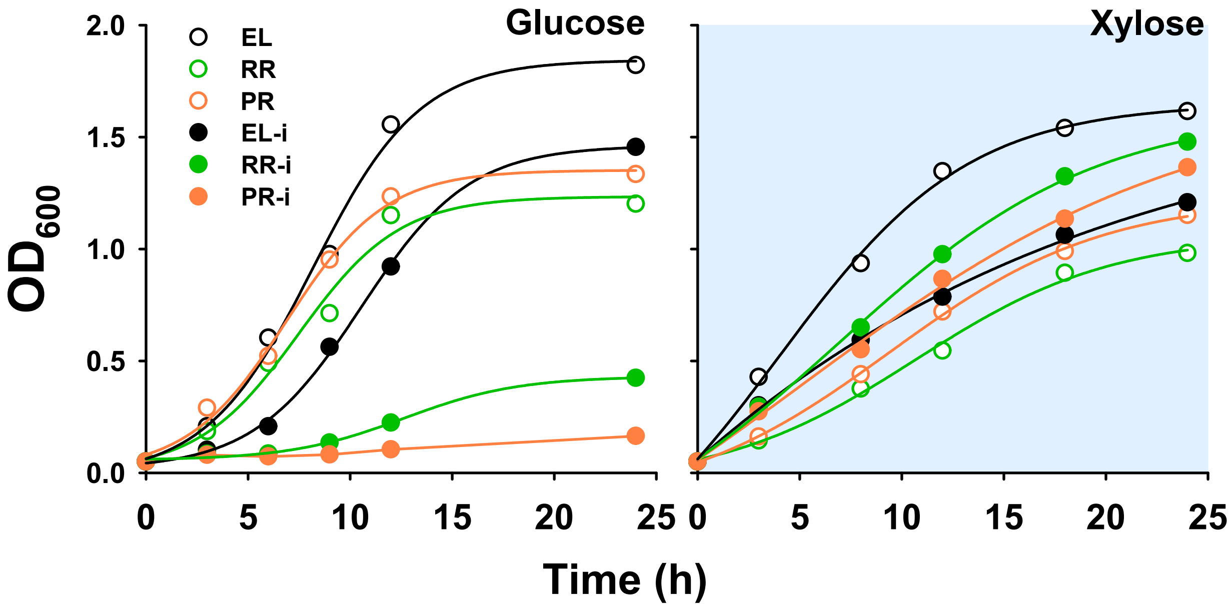

Figure S4 Effect of knockdown *pfk*AB and *zwf* gene using CRISPRi (i.e., shown by "i" mark) on the growth of recombinant ET7 strains under glucose- and xylose-based medium. Strains were cultured using baffled flasks in the regular incubator without CO_2 supply at 37°C**.**

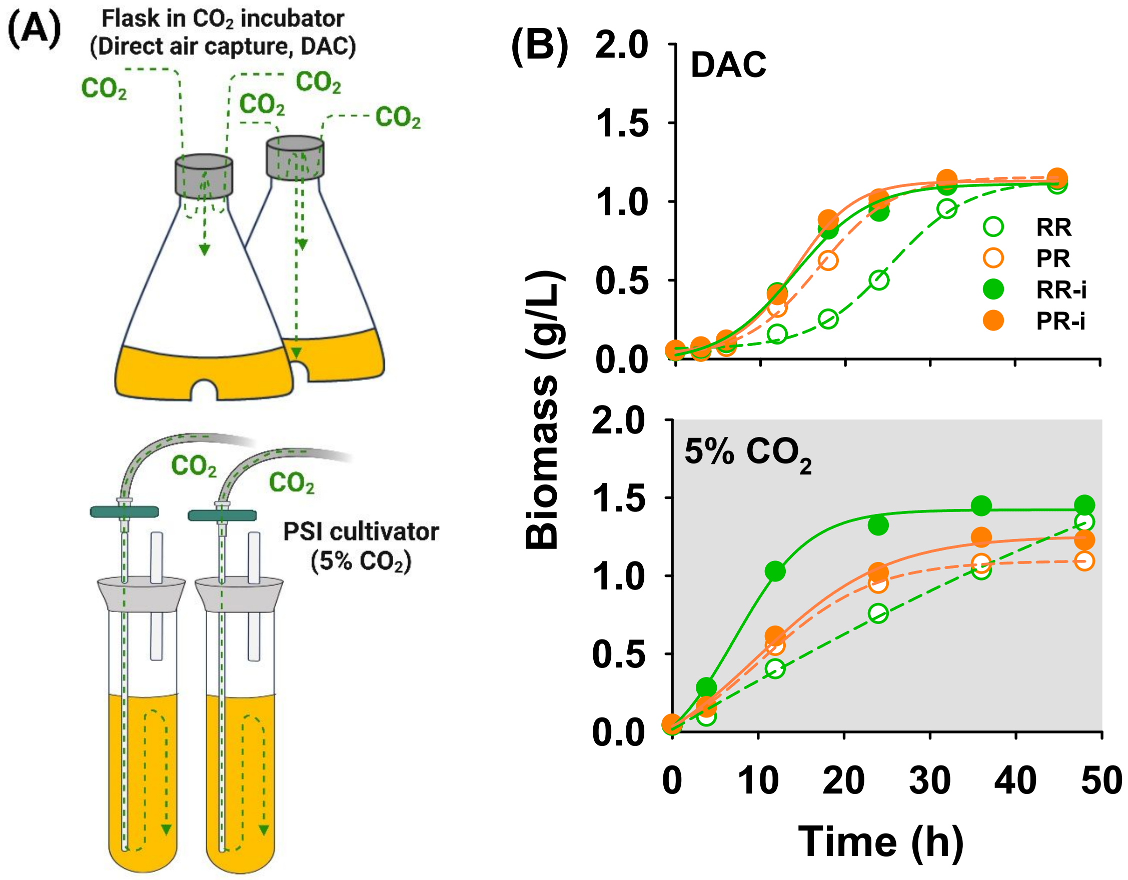

Figure S5 (A) Illustration of cultural devices used with different supplementation of CO_2 levels including direct CO_2 capture from the air (DAC) and PSI multi-cultivator with 5% CO_2 supply. **(B)** Effect of knockdown *pfk*AB and *zwf* gene using CRISPRi (i.e., shown by "i" mark) on the growth of recombinant ET7 strains under xylose-based medium at different CO_2 levels. DAC indicates direct CO_2 capture from air which was supplied in the incubator and 5% CO_2 was cultured using PSI cultivator with a flow rate of 40 mL/min CO_2. Strains were cultured using a baffled flask at 37°C.

Table S1 Metabolites profile including remnant carbon and accumulated compounds of engineered ET7 strains after 32 h cultivation in xylose medium with DAC and 5% CO_2 supply.

Strain	Remnant carbon* (g/L)		Accumulated compounds (g/L)		
	xylose	5-ALA	lactate	acetate	succinate
**DAC**					
Rc-i	1.57	0.49	0.10	1.33	0.65
ARc-i	0.00	0.89	0.00	0.90	0.50
APc-i	0.00	1.14	0.10	0.82	0.20
**5% CO_2**					
Rc-i	1.24	0.58	0.54	1.49	1.05
ARc-i	0.80	1.26	0.42	1.55	0.91
APc-i	1.50	1.66	0.29	1.18	0.60

*Carbon input is 10 g/L of xylose.

Table S2 Elemental analysis and biomass quantification of engineered ET7 strains after 32 h cultivation in xylose medium with DAC and 5% CO_2 supply.

**Strains**	**Average element portion (%)**			**Dry cell weight (g/L/OD)**	**OD_{600}**	**$C_{Biomass}$* (g/L)**
	**carbon**	**nitrogen**	**hydrogen**			
			**DAC**			
Rc-i	45.75	12.9	7.131	0.50	1.83	0.42
ARc-i	46.26	13.61	7.05	0.68	2.54	0.80
APc-i	45.93	13.19	7.04	0.67	2.05	0.63
			**5% CO_2**			
Rc-i	43.83	11.45	6.76	0.59	1.52	0.39
ARc-i	46.91	13.58	7.12	0.66	3.03	0.94
APc-i	44.52	13.35	6.95	0.64	2.63	0.75

*Biomass = carbon content x dry cell weight x OD_{600} (i.e., optical density of cell growth at 24 h.

Table S3 Calculation of CO_2 assimilation capability in engineered ET7 strains using xylose medium with DAC and 5% CO_2 supply after 32 h.

**Strains**	**C $_{metabolites}$** (Coefficient $_{carbon}$[a] x metabolites[b])					**Specific CO_2 assimilation** [c]
	**xylose**	**lactate**	**acetate**	**succinate**	**5-ALA**	g-CO_2/g-5-ALA
			**DAC**			
Rc-i	0.63	0.04	0.32	0.68	0.22	-13.94
ARc-i	0.00	0.00	0.04	0.41	0.41	-9.19
APc-i	0.00	0.04	0.32	0.00	0.52	-7.78
			**5% CO_2**			
Rc-i	0.50	0.22	0.60	0.43	0.31	-10.57
ARc-i	0.32	0.17	0.62	0.37	0.58	-3.07
APc-i	0.60	0.12	0.47	0.47	0.76	-2.42

[a] Coefficient carbon is calculated from carbon content in the chemical formula.

[b] Metabolites are defined from either remnant carbon or accumulated compounds.

[c] The specific carbon dioxide assimilation is calculated by $([C_{biomass}] + [C_{metabolites}] - [C_{in}^{d}]) \times \frac{1}{ALA} \times \frac{1}{0.27}$ in the unit of g-CO_2/g-ALA.

[d] C_{in} is determined as: Coefficient $_{carbon}$ x carbon input in g/L unit (i.e., sole carbon used is 10 g/L). C_{in} of xylose is 4.

Table S4 The strains, plasmids, primers and DNA sequences used in this study.

Materials	Description	Remark
**Strains**		
DH5α	F⁻ *endA1 glnV44 thi-1 recA1 relA1 gyrA96 deoR nupG purB20* φ80d *lacZ*△M15 △(*lacZYA-argF*) U169, *hsd*R17($r_K^- m_K^+$), λ^-	NEB
MG1655	F^-, lambda-, rph^{-1}	Lab stock
EcN	Wild type of *E. coli* Nissle 1917	Lab stock
MT7L	MG1655 integrated with T7 RNA polymerase under LacUV5 promoter at lambda site	This study
ET7L	Nissle 1917 integrated with T7 RNA polymerase under LacUV5 promoter at lambda site	This study
**Plasmids**		
p21a-R15PI-RuBisCO (RR)	pMB1 ori, Cm^R, lacI, T7-1 promoter, T7OS RBS, rbcLS from *Synechococcus elongatus* PCC6301, T7-2 promoter, B0034 RBS, ribose 1,5-bisphosphate isomerase (R15PI) from *Thermococcus kodakarensis*	This study
p21a-PRK-RuBisCO (PR)	pMB1 ori, Cm^R, lacI, T7-1 promoter, T7OS RBS, rbcLS from *S. elongatus* PCC6301, T7-2 promoter, B0034 RBS, phosphoribokinase (PRK) from *S. elongatus* PCC6301	This study
pSIT-Rc (Rc)	RSF ori, Km^R, lacI, single T7 promoter, T7OS RBS, ALAS from *Rhodobacter capsulatus* (Rc)	(24)
pSIT-Rc-RR (ARc)	RSF ori, Km^R, lacI, T7-1 promoter, T7OS RBS, RcALAS, T7-2 promoter, T7OS RBS, rbcLS from *S. elongatus* PCC6301, B0034 RBS, PRK from *S. elongatus* PCC6301	This study
pSIT-Rc-PR (APc)	RSF ori, Km^R, lacI, T7-1 promoter, T7OS RBS, RcALAS, T7-2 promoter, T7OS RBS, rbcLS from *S. elongatus* PCC6301, B0034 RBS, R15PI from *T. kodakarensis*	This study
DRc	Dual plasmids of RR and Rc	This study
DPc	Dual plasmids of PR and Rc	This study
dCas9-3PSG (i)	CloDF13 ori, Spc^R, lacI, synthetic promoter-1, *pfk*APsg, synthetic promoter-2, *pfk*BPsg, synthetic promoter-3, *zwf*Psg, dCas9	This study
Rc-i	Dual plasmids of Rc and dCas9-3PSG	This study
ARc-i	Dual plasmids of ARc and dCas9-3PSG	This study
APc-i	Dual plasmids of APc and dCas9-3PSG	This study

Primers		
BamHI-B0034-HindIII-R15Pi-F	GAGGATCCAAAGAGGAGAAAAAGCTTATGGCCGTGGTGAAGGAGGTGCTTGAAAT	This study
PstI-SpeI-R15Pi-R	TCCTGCAGACTAGTTCAGTCTTCCCACGGCTCAGTATA	This study
NdeI-BamHI-rbcLS-F	TACATATGGGATCCATGCCCAAGACGCAATCTGC	This study
NotI-rbcLS-R	ATGCGGCCGCTTAGTAGCGGCCGGGACGATGAA	This study

DNA sequences of CO_2-fixing genes

R15PI from *Thermococcus kodakarensis*

ATGGCCGTGGTGAAGGAGGTGCTTGAAATTGCTGAAAAAATTAAGAATATGGAAA
TCCGTGGCGCCGGAAAGATCGCTCGTTCCGCCGCTTATGCACTTCAGCTTCAGGCG
GAAAAGTCTAAAGCTACGAATGTCGACGAGTTTTGGAAAGAGATGAAACAAGCC
GCTAAGATTTTGTTTGAAACCCGTCCGACGGCGGTTTCGTTACCAAATGCGTTACG
TTATGTGATGCACCGTGGCAAAATTGCGTATTCGAGCGGTGCTGACCTTGAACAA
CTTCGTTTTGTTATTATTAACGCCGCGAAAGAATTTATTCACAACTCGGAGAAAGC
TCTGGAGCGTATCGGCGAGTTTGGCGCGAAGCGTATTGAAGATGGCGATGTCATC
ATGACACACTGCCATTCCAAGGCGGCTATCTCTGTCATGAAAACAGCGTGGGAAC
AGGGCAAAGATATTAAAGTGATCGTCACCGAGACACGCCCGAAGTGGCAGGGTA
AAATCACCGCAAAGGAACTGGCCTCGTACGGTATCCCTGTGATCTATGTGGTCGA
TTCAGCAGCACGCCACTATATGAAAATGACAGACAAGGTTGTGATGGGCGCAGAC
TCTATTACCGTTAATGGGGCAGTCATCAATAAGATTGGGACCGCCTTGATCGCATT
AACTGCGAAAGAACACCGCGTCTGGACAATGATCGCAGCGGAAACGTATAAATTC
CATCCCGAGACGATGTTAGGGCAACTTGTAGAAATTGAGATGCGCGACCCGACAG
AAGTAATTCCTGAAGATGAGCTGAAAACCTGGCCCAAGAATATTGAGGTTTGGAA
CCCGGCTTTCGATGTGACCCCACCTGAGTACGTGGACGTTATTATTACCGAACGTG
GGATCATCCCCCCGTATGCAGCCATTGATATCTTACGTGAAGAATTTGGGTGGGCA
TTGAAATATACTGAGCCGTGGGAAGACTGA

RuBisCO (*rbc*LS)

*rbc*L from *Synechococcus elongatus* PCC6301

ATGCCCAAGACGCAATCTGCCGCAGGCTATAAGGCCGGGGTGAAGGACTACAAA
CTCACCTATTACACCCCCGATTACACCCCCAAAGACACTGACCTGCTGGCGGCTTT
CCGCTTCAGCCCTCAGCCGGGTGTCCCTGCTGACGAAGCTGGTGCGGCGATCGCG
GCTGAATCTTCGACCGGTACCTGGACCACCGTGTGGACCGACTTGCTGACCGACA
TGGATCGGTACAAAGGCAAGTGCTACCACATCGAGCCGGTGCAAGGCGAAGAGA
ACTCCTACTTTGCGTTCATCGCTTACCCGCTCGACCTGTTTGAAGAAGGGTCGGTC
ACCAACATCCTGACCTCGATCGTCGGTAACGTGTTTGGCTTCAAAGCTATCCGTTC
GCTGCGTCTGGAAGACATCCGCTTCCCCGTCGCCTTGGTCAAAACCTTCCAAGGTC
CTCCCCACGGTATCCAAGTCGAGCGCGACCTGCTGAACAAGTACGGCCGTCCGAT
GCTGGGTTGCACGATCAAACCAAAACTCGGTCTGTCGGCGAAAAACTACGGTCGT
GCCGTCTACGAATGTCTGCGCGGCGGTCTGGACTTCACCAAAGACGACGAAAACA
TCAACTCGCAGCCGTTCCAACGCTGGCGCGATCGCTTCCTGTTTGTGGCTGATGCA
ATCCACAAATCGCAAGCAGAAACCGGTGAAATCAAAGGTCACTACCTGAACGTGA
CCGCGCCGACCTGCGAAGAAATGATGAAACGGGCTGAGTTCGCTAAAGAACTCGG
CATGCCGATCATCATGCATGACTTCTTGACGGCTGGTTTCACCGCCAACACCACCT
TGGCAAAATGGTGCCGCGACAACGGCGTCCTGCTGCACATCCACCGTGCAATGCA
CGCGGTGATCGACCGTCAGCGTAACCACGGGATTCACTTCCGTGTCTTGGCCAAGT
GTTTGCGTCTGTCCGGTGGTGACCACCTCCACTCCGGCACCGTCGTCGGCAAACTG
GAAGGCGACAAAGCTTCGACCTTGGGCTTTGTTGACTTGATGCGCGAAGACCACA
TCGAAGCTGACCGCAGCCGTGGGGTCTTCTTCACCCAAGATTGGGCGTCGATGCC
GGGCGTGCTGCCGGTTGCTTCCGGTGGTATCCACGTGTGGCACATGCCCGCACTGG
TGGAAATCTTCGGTGATGACTCCGTTCTCCAGTTCGGTGGCGGCACCTTGGGTCAC
CCCTGGGGTAATGCTCCTGGTGCAACCGCGAACCGTGTTGCCTTGGAAGCTTGCGT
CCAAGCTCGGAACGAAGGTCGCGACCTCTACCGTGAAGGCGGCGACATCCTTCGT
GAAGCTGGCAAGTGGTCGCCTGAACTGGCTGCTGCCCTCGACCTCTGGAAAGAGA
TCAAGTTCGAATTCGAAACGATGGACAAGCTCTAA

rbcLS from *Synechococcus elongatus* PCC6301

ATGAGCATGAAAACTCTGCCCAAAGAGCGTCGTTTCGAGACTTTCTCGTACCTGCC
TCCCCTCAGCGATCGCCAAATCGCTGCACAAATCGAGTACATGATCGAGCAAGGC
TTCCACCCCTTGATCGAGTTCAACGAGCACTCGAATCCGGAAGAGTTCTACTGGAC
GATGTGGAAGCTCCCCCTGTTTGACTGCAAGAGCCCTCAGCAAGTCCTCGATGAA
GTGCGTGAGTGCCGCAGCGAATACGGTGATTGCTACATCCGTGTCGCTGGCTTCG
ACAACATCAAGCAGTGCCAAACCGTGAGCTTCATCGTTCATCGTCCCGGCCGCTA
CTAA
